# Hybrid Percutaneous Brachiofemoral Shunt and Open Abdominal Aortic Aneurysm Repair in a Kidney Transplant Recipient

**DOI:** 10.1155/2021/6655660

**Published:** 2021-07-31

**Authors:** Javad Salimi, Sayed Alimohammad Sadat, Mohammad Javad Yavari Barhaghtalab, Hormat Rahimzadeh

**Affiliations:** ^1^Trauma and Surgery Research Center, Sina Hospital, Tehran University of Medical Science, Tehran, Iran; ^2^Department of General Surgery, Shahid Beheshti Hospital, Yasuj University of Medical Science, Yasuj, Iran; ^3^Department of Internal Medicine, Sina Hospital, Tehran University of Medical Sciences, Tehran, Iran

## Abstract

Abdominal aortic aneurysm (AAA) repair in kidney transplant recipients may cause ischemia in the transplanted kidney. As a result, various techniques have been described for protection of the renal allograft during AAA repair including temporary shunt, extracorporeal bypass, cold renal perfusion, endovascular aortic aneurysm repair (EVAR), and operation without renal allograft protection. We successfully treated a 56-year-old man, a case of kidney transplantation with AAA, using a temporary hybrid percutaneous brachiofemoral shunt using vascular prosthesis with a long 7-French (Fr) catheter sheath introducer (CSI) in the aortic arch via the right brachial artery and 8 Fr CSI in the right femoral artery that were connected together with a 7 Fr guiding catheter, before aortic cross-clamping and repair of AAA using a Dacron tube graft. The patient recovered well from the surgery without any complication and was discharged on the 6^th^ postoperative day. To our knowledge, this is the first report of using a temporary hybrid percutaneous brachiofemoral shunt for renal allograft protection in AAA repair surgery in a patient with kidney transplantation, and we think that this temporary shunt is an easy, safe, and rapid method for renal allograft protection from ischemia.

## 1. Background

An arterial aneurysm is defined as a focal dilation of a blood vessel with respect to the original artery. AAA may be detected incidentally or at the time of rupture. The likelihood that an aneurysm will rupture is influenced by the aneurysm size expansion rate, continued smoking, and persistent hypertension. Treatment is recommended when it reaches 5 cm to 5.5 cm, is demonstrated as rapidly enlarging >0.5 cm over 6 months, or becomes symptomatic. Open surgical repair via a transabdominal or retroperitoneal approach has been the gold standard [1, 2].

With increasing graft survival after kidney transplantation and high rates of cardiovascular risk factors in renal transplant recipients, vascular surgeons will increasingly be faced with repairing AAAs in this patient group. It is important to avoid ischemic and reperfusion injury to the transplanted kidney. In high-risk patients, collateral perfusion reduces the risk of ischemic complications when prolonged infrarenal aortic cross-clamping is probable in open aortic and iliac aneurysm repair surgery [3–6].

Various techniques reported in literatures for decreasing the ischemic effect of aortic clamping during AAA repair are summarized in Table 1.

In this study, we report an open AAA repair surgery in a kidney transplant patient who was successfully operated with a temporary hybrid percutaneous brachiofemoral shunt using vascular prosthesis.

## 2. Case Presentation

**Figure 1:**
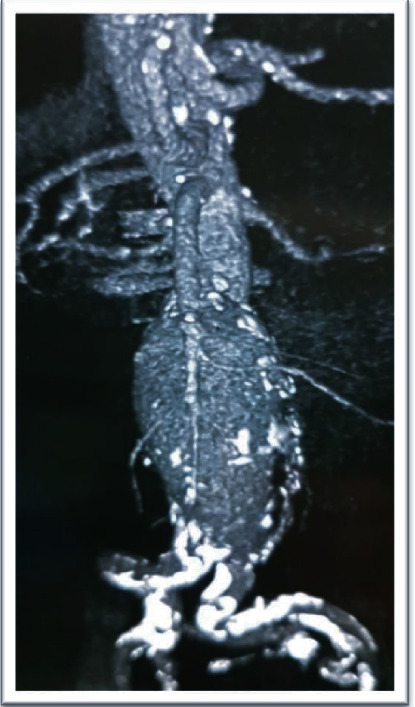
Abdominopelvic CT scan without contrast before AAA repair operation.

**Figure 2:**
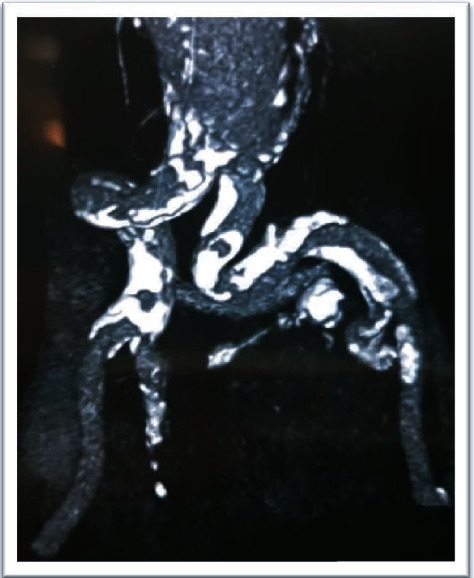
Abdominopelvic CT scan without contrast before AAA repair operation.

**Figure 3:**
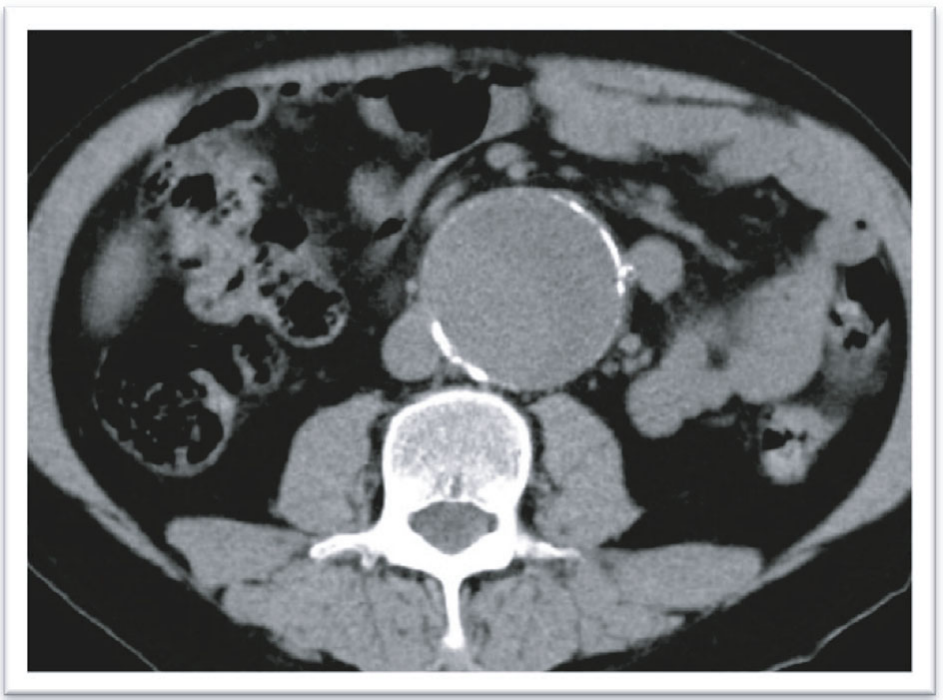
Abdominopelvic CT scan without contrast before AAA repair operation.

A 56-year-old man with end-stage renal disease (ESRD) secondary to membranous glomerulonephritis had undergone a right-sided orthotopic renal transplantation 4 months earlier after 1.5 years of hemodialysis. His renal function was pretty good after transplantation, and he had been discharged with serum creatinine 1.6 mg/dl and had been stable over these 4 months. Renal functional scan (Tc-99m DTPA (diethylene-triamine-pentaacetate)) was normal. Past medical history was positive for hypertension, hyperlipidemia, and coronary artery disease (three-vessel disease). The patient had an AAA with an anterioposterior diameter of 4.2 cm and symptom-free, so vascular surgery consultation was not done before renal transplantation. He was receiving tacrolimus, mycophenolic acid, and prednisolone after the transplantation. The patient developed with abdominal pain and raising serum creatinine (2.6 mg/dl), and abdominopelvic sonography revealed normal transplanted kidney with a large infrarenal abdominal aortic aneurysm (anterioposterior diameter of 55 mm) which did not involve the iliac arteries, and then, computed tomography (CT) scan without contrast confirmed the diagnosis (Figures 1–3).

**Figure 4:**
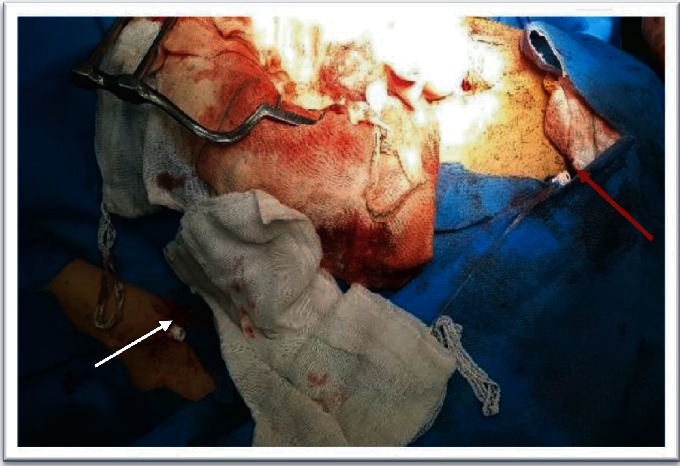
Intraoperative photography of the brachial sheath (white arrow) and femoral sheath (red arrow) used for brachiofemoral shunt during the operation.

After appropriate hydration and medical therapy and lowering creatinine to 1.7 mg/dl, the patient was scheduled for an operation. As both iliac arteries were tortuous and calcified, they were unsuitable for the EVAR. For renal allograft protection, at first, a short 5 Fr CSI (Cordis AVANTI®+ Sheath Introducer, USA) was inserted in the right brachial artery, and then, a 65 cm 7 Fr percutaneous CSI (Epsylar-Optimed, Germany) was placed in the aortic arch under fluoroscopy on the guide of a stiff wire (Amplatz wire, Cordis, USA) via the brachial access, and an 11 cm 8 Fr CSI (Cordis AVANTI®+ Sheath Introducer, USA) was inserted in the right external iliac via the common femoral artery through a percutaneous procedure and was fixed (Figure 4).

After laparotomy and dissecting of the aorta up to the renal artery and down to both the common iliac arteries and intravenous injection of 5000 international units (IU) of unfractionated heparin, connection between both sheaths was established with a 7 Fr guiding catheter (its end was cut) and connection was covered with sterile towels and protected from compression and kinking. After confirmation of the good function of the shunt, aortic cross-clamping was done and the aneurysm was repaired with knitted Dacron prostheses 20 × 10 mm (Jotec GmbH, Hechingen, Germany) in about 35 minutes. To maintain the flow to both the lower extremity and the transplanted kidney (proximally), the common iliac artery was clamped as the end of the sheath was in the external iliac artery. Hydration was done before and during the operation, and two units of packed red blood cells were transfused. Adequate urine output of 30 cc per hour was maintained during the operation. At the end of the operation, brachial and femoral sheaths were removed, and no closure device was used.

The patient was transferred to the surgical intensive care unit (ICU) and was extubated after 4 hours. The patient developed atrial fibrillation that was controlled with intravenous amiodarone at the first day postoperation. The patient was transferred to the post-ICU vascular surgery ward at the 3^rd^ day postoperation. There were no any other complications in the postoperative period, and the patient was discharged in stable vital sign and good condition with creatinine 1.6 mg/dl at the 6^th^ day postoperation. The patient had no complaint during the 18-month clinical follow-up, and the last serum creatinine was 1.7 mg/dl.

## 3. Discussion and Conclusion

The AAA in a kidney-transplanted patient requires surgical management and is truly a life-threatening condition and has a more complicated repair. The transplanted kidney has a solitary arterial supply and lacks collateral circulation, so it is more susceptible to ischemic injury, so aortic cross-clamping may put the renal allograft at risk for ischemic damage [11]. If the period of aortic cross-clamping in open surgery is less than 50 min, slight protection may be needed and a clamp and go method could be done [6]. To minimize this risk, as was said before, there is a variety of techniques to protect the transplanted kidney in major open aortoiliac surgeries, especially AAA repair [3–11].

A different renal protecting mechanism could be the endovascular repair. The benefit is that the allograft ischemia is limited to a short time that the graft is ballooned into place, but the disadvantages are the emboli to the kidney from dislodged atherosclerotic debris during the procedure as well as the risk of contrast-induced nephropathy [7, 11].

This report is the first report of percutaneous brachiofemoral shunt for ischemic protection of the transplanted kidney in AAA repair. We think that despite the fact that this shunt can support collateral perfusion to the transplanted kidney during operation. This protective shunt is easy and safe and decreases total time of operation that is important in high-risk patients such as our patient with three-vessel coronary disease and high serum creatinine. It is obvious that this procedure needs to be developed and uses a larger shunt.

In summary, when we are not sure about the time of operation and aortic clamping and if the patient is at the high risk for operation, it is better to use protective measures to decrease ischemic effect on the transplanted kidney. This percutaneous temporary shunt is an easy and rapid way for the ischemic protection of the transplanted kidney during AAA repair or other major aortoiliac procedures.

## Figures and Tables

**Table 1:** Techniques used for protection of the transplanted kidney during AAA repair.

Femoral V-A bypass [4]
Pump-oxygenation bypass technique [5]
Axillofemoral shunt [5, 6, 8]
Atriofemoral bypass [7]
Endovascular aneurysm repair (EVAR) [5, 7]
Topical cooling [5, 7]
Aortofemoral shunt [8]
Femorofemoral bypass with extracorporeal circulation [6–8]
Operation without renal allograft protection [9]
Femoropoliteal bypass [9]
Permanent and temporary axillofemoral bypass [7–9]
Saphenous vein branchiorenal shunt [10]
Aortoiliac shunt [7, 10]
Cold perfusion of the graft [7–9, 11]
General hypothermia [8, 9, 11]

## Data Availability

The datasets used and/or analyzed during the current study are available from the corresponding author on reasonable request.

## References

[1] Aggarwal S., Qamar A., Sharma V., Sharma A. (2011). Abdominal aortic aneurysm: a comprehensive review. *Experimental and Clinical Cardiology*.

[2] Shaw P. M., Loree J., Gibbons R. C. (2020). Abdominal aortic aneurysm. *StatPearls [Internet]*.

[3] Dregelid E. (2014). Temporary extracorporeal brachio-femoral vascular prosthesis shunt for ischemia prevention in an operation for abdominal aortic and iliac aneurysms in a patient with Marfan^|^apos;s syndrome. *Annals of Thoracic and Cardiovascular Surgery*.

[4] Maeda T., Watanabe N., Muraki S. (2009). Abdominal aortic aneurysm repair in a renal transplant recipient using a femoral V-A bypass. *Annals of Thoracic and Cardiovascular Surgery*.

[5] Callaghan C. J., Munday I. T., Casey N. D., Large S. R., Gaunt M. E. (2005). Abdominal aortic aneurysm repair in a renal transplant recipient: a modified pump-oxygenation bypass technique to reduce hypotension and myocardial ischaemia. *EJVES Extra*.

[6] Sadat U., Huguet E. L., Varty K. (2010). Abdominal aortic aneurysm surgery in renal, cardiac and hepatic transplant recipients. *European Journal of Vascular and Endovascular Surgery*.

[7] Joh J. H., Nam D. H., Park H. C. (2013). Endovascular abdominal aortic aneurysm repair in patients with renal transplant. *Journal of the Korean Surgical Society*.

[8] Lepäntalo M., Biancari F., Edgren J., Eklund B., Salmela K. (1999). Treatment options in the management of abdominal aortic aneurysm in patients with renal transplant. *European Journal of Vascular and Endovascular Surgery*.

[9] Harris J. P., May J. (1987). Successful aortic surgery after renal transplantation without protection of the transplanted kidney. *Journal of Vascular Surgery*.

[10] Khan R., Simms M. (2005). Renal protection strategies when aortic aneurysm repair necessitates renal artery re-implantation: review and a new technique. *The Internet Journal of Surgery*.

[11] Kim H. K., Ryuk J. P., Choi H. H., Kwon S. H., Huh S. (2009). Abdominal aortic aneurysm repair in patient with a renal allograft: a case report. *Journal of Korean Medical Science*.

